# Effect of xylo-oligosaccharides on reproduction, lipid metabolism, and adipokines of hens during the late egg-laying period

**DOI:** 10.5713/ab.22.0049

**Published:** 2022-06-24

**Authors:** Fengyun Wen, Fengyan Wang, Pei Li, Hanyi Shi, Ning Liu

**Affiliations:** 1 Department of Animal Science, Henan University of Science and Technology, Luoyang 471023, China; 2 Department of Poultry Science, University of Georgia, Athens, GA 30605, USA

**Keywords:** Adipokine, Hens, Lipid Metabolism, Reproducibility, Xylo-oligosaccharides

## Abstract

**Objective:**

The present study aimed to investigate the effect of xylo-oligosaccharides (XOS) administration on egg production, reproductive hormones, serum lipids and adipokines of hens at the late cycle of reproduction.

**Methods:**

Four treatments included control (basal diet) and XOS addition at 2.0 (XOS-2), 4.0 (XOS-4), or 6.0 (XOS-6) g/kg of diet using 288 commercial Hy-Line brown hens from 73 to 84 wk of age. Egg production, body fat deposition, reproductive tract and hormones, lipid metabolism and adipokines were determined.

**Results:**

At 84 wk, compared to the control, XOS supplementation at the three doses increased (p<0.001) egg-laying rates by 13.2% averagely, which led to a higher egg mass by 131 g/hen throughout the whole trial period. Abdominal fat and skinfold of XOS treatments were decreased (p<0.001) by 26.1% and 18.6%, respectively; large follicles and ovary weight were increased (p<0.001) by 0.73 follicle/hen and 18.6%, respectively. For serum parameters, cholesterol and triglyceride were decreased (p<0.001) by 17.5% and 29.2%, respectively; luteinizing hormone, follicle-stimulating hormone, and progesterone were increased (p≤ 0.001) by 16%, 31%, 29%, respectively; adiponectin and visfatin were increased (p<0.001) by 34% and 44%, respectively; but chemerin and leptin were decreased (p≤0.001) by 22% and 14%, respectively. With the increased XOS doses, linear decreases (p<0.05) were found on abdominal skinfold and serum triglyceride.

**Conclusion:**

The obtained data indicate that XOS can be used as an additive to improve fecundity by beneficially modulating fat deposition, lipid metabolism, reproductive hormones, and adipokines of hens at the late cycle of reproduction.

## INTRODUCTION

Xylo-oligosaccharides (XOS) are polymers of two to nine xylose units degenerated from the xylan fraction in plant fiber. It is well documented that the XOS acts as a prebiotic selectively feeding beneficial bacteria within the digestive tract, and the alterations in the microbiota might be further involved in metabolism and cell signaling. Indeed, latest studies reveal that XOS can alleviate hepatic steatosis by improving hepatic metabolism and sterol excretion in rodents [1,2]. Similarly, in pigs, XOS decreased heptane diamine and increased short-chain fatty acids [3].

Moreover, XOS was claimed effective on the down-regulation of adipose deposition in obese models [4,5]. As known, obesity is modern man’s fertility nemesis and so of farm animals, especially hens [6,7]. Modern strains of laying hens, commercial or breeding, have a strong natural propensity to body fat deposition with age, which is the main reason for the gradual decline in health and reproduction besides the natural physiological factor [8,9]. Consequently, dietary manipulation to avoid early aging or lengthening laying cycle is becoming a research focus for poultry nutritionists and producers.

The fertility of hens is controlled by hormones mainly including luteinizing hormone (LH), follicle-stimulating hormone (FSH), and progesterone (Prog). The obesity is highly related to adipocytokine signals, or adipokines, such as adiponectin, visfatin, chemerin, and leptin. These adipokines also have a critical role in reproductive functions [10]. However, it is unclear about the XOS effect on reproductive hormones and adipokines in both clinics and animals. Therefore, based on the confirmed effect of XOS on lipid metabolism and obesity, it is curious whether these effects act as a cascade influence the female fertility.

The hypothesis of the present study is that XOS can improve the reproducibility of aged hens by regulating reproductive hormones and adipose toxicity. The aim of present study was to investigate the effect of dietary XOS added at 2 to 6 g/kg on the fecundity, reproductive system, and hormones, as well as adipokines of hens during the late phase of laying cycle.

## MATERIALS AND METHODS

### Animal ethics approval

The trial protocol was approved by the Institutional Committee for Animal Use and Ethics of the College of Animal Science in Henan University of Science and Technology (No. 2018022).

### Xylo-oligosaccharides and treatments

The XOS additive was produced from corn cobs with a chemical profile of XOS 70.7%, xylose 20.3%, glucose 3.2%, acetic acid 0.2%, and others 5.6%. The XOS was purchased from Shandong Longlive Bio-technology Co., Ltd (Jinan, China). There were four treatments with XOS addition at 0 (Control, basal diet), 2.0 (XOS-2), 4.0 (XOS-4), or 6.0 (XOS-6) g/kg of diet at the expense of corn. The 6.0 g/kg of XOS can cause a metabolizable energy difference of 19.2 kcal/kg, which is neglected. All diets were formulated as isoenergy and isonitrogen. The XOS doses were established by the description of the product and the pilot trial of the authors. The basal diet (Table 1) was formulated based on corn and soybean meal, with compositions and nutrient levels referred to Hy-Line brown laying hens and local practice. All diets were fed in a mash form. To prepare the experimental diet, the maximum and minimum concentrations of the experimental diet were first mixed separately and then used to prepare the other experimental concentrations.

### Animals and samples

A total of 288 commercial Hy-Line brown hens at 72 wk of age with similar egg production performance, body weights, and abdominal skinfolds were randomly assigned to four dietary groups. Each treatment contained 6 replicates and 12 hens per replicate. All replicates were uniformly distributed in a chicken house with three tier battery cages to minimize the environmental effect. All hens were supplied with feed at 108 g/hen/day and *ad libitum* water according to the management manual of Hy-Line brown strain in the house with auto-ventilation, lighting system at 16 L/8 D, and the temperature at approximately 22°C [11]. The feeding trial lasted from 73 to 84 wk of age. Feed intake, egg laying rate, and egg mass per replicate were recorded daily.

At the last day of 72, 76, 80, and 84 wk, all hens per replicate were bled from their wing vein for the preparation of serum [12]. At 84 wk, skinfold thickness of all birds was measured by longitudinally grabbing up skins located at the 2 cm site of abdominal sternum nodule using a skinfold caliper (HeartWell Medical Supplies, Lakewood, NJ, USA). After the body measurement, hens were suffocated by carbon dioxide and dissected for weighing liver, abdominal fat, ovary, and oviduct, and numbering follicles with a diameter ≥1 cm including eggs in the oviduct. The index of organ weight and body adiposity were expressed as a percentage of body weight to minimize the error resulted from individual body weight.

### Chemical and biochemical analysis

Compositions of XOS were analyzed by high performance liquid chromatography after diluted-acid hydrolysis according to the method described by China National Standard GB/T 35545-2017 [13]. Chemical analysis of proximate nutrients in diets was carried out according to the method by Liu et al [14]. For serum lipids and reproductive hormones, assay kits were purchased from Nanjing Jiancheng Bioengineering institute (Nanjing, China) for total cholesterol (A111-1-1), triglycerides (A110-1-1), Prog (Prog, H089), LH (H206), follicle stimulating hormone (FSH, H101). The sample preparation and testing processes were referred to the specification of assay kits and the previous methods of authors [14].

Serum concentration of adipokines was determined using chicken-specific enzyme linked immunosorbent assay kits from Antibodies-online (Limerick, PA, USA) for leptin (ABIN6957434, sensitivity 13.1 pg/mL), adiponectin (ABIN 6953507, sensitivity 49 pg/mL), and chemerin (ABIN1051892, sensitivity 50 pg/mL) with an intra-assay CV<10% and inter-assay CV<12%. Visfatin kit (LS-F49064) was from LSBio (Seattle, WA, USA) with the sensitivity 1.563 ng/mL, intra-assay and inter-assay CV<10%. Serum samples were equally pooled by replicate and detected in triplicate following the manufacturer’s protocols.

### Statistical analysis

Data in tables and figures are represented as mean and standard error of the mean using SAS software (SAS 9.4, Cary, NC, USA). Differences between mean values of normally distributed data were assessed with one-way analysis of variance by Tukey’b-test and data with heterogeneity variance and follicle number by nonparametric Tamhane’s T2 test at p<0.05 level of significance. The average mean per replicate was the statistical unit for the parameters of egg production, hormones, and lipid metabolism. The trend of XOS doses at 2.0, 4.0, and 6.0 g/kg for each experimental parameter was analyzed using linear and quadratic polynomial contrasts.

## RESULTS

### Xylo-oligosaccharides and egg production

**Figure 1:**
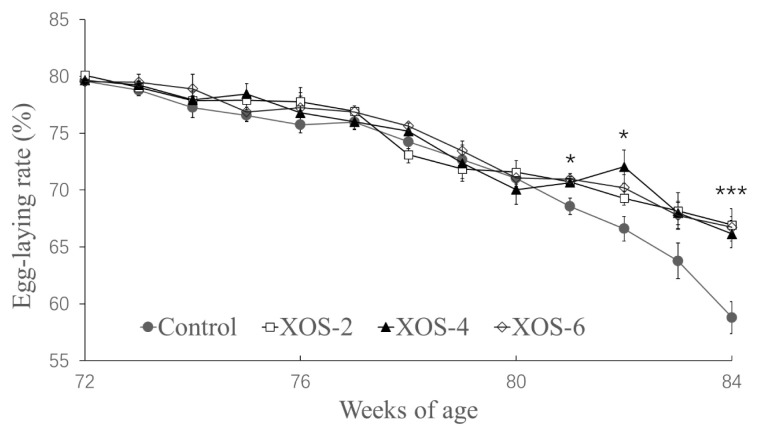
Effect of xylo-oligosaccharides (XOS) on the egg-laying rate of Hy-Line hens. XOS-2, XOS-4, and XOS-6, containing XOS at 2.0, 4.0 and 6.0 mg/kg of diet. * p<0.05 and *** p<0.001, treatment effect by Turkey’s-b test.

In Figure 1, diets supplemented with XOS at the three doses influenced egg-laying rates at 78 (p = 0.030), 81 (p = 0.013), 82 (p = 0.017) and 84 wk (p<0.001). In detail, at 78 wk, the egg-laying rate of XOS-6 was greater than that of XOS-2, but there were no statistical differences between the control and the XOS treatments; at 82 wk, the egg-laying rate of XOS-4 was greater than that of control, but the other XOS doses had no differences from the control; at 81 and 84 wk, the egg-laying rates of the three doses of XOS were higher than that of the control.

In contrast with the control, the dietary treatments also influenced the grams of egg mass as of wk 73 to 84 (p<0.001), which reached an average increase of 131 g/hen (Table 2), but there were no differences among the three XOS doses. The dose trends by linear or quadratic analyses were also not significant for the accumulated egg mass.

### Xylo-oligosaccharides and adipose deposition

As shown in Table 2, the dietary treatment effects were significant on the liver weight percentages (p = 0.041), abdominal fat percentages (p<0.001), and skinfolds (p<0.001). Compared to the control, the liver weight percentage was decreased (p<0.05) in XOS-6, but not in other doses. Abdominal fat percentages of hens fed XOS at the three doses were decreased (p<0.05) compared to the control, and the effect of XOS-6 was more pronounced, but there were no differences among the three doses.

Similarly, abdominal skinfolds were lowered (p<0.05) in the XOS treatments, which were lower (p<0.05) in XOS-4 and XOS-6 than XOS-2, but there was no difference between XOS-4 and XOS-6. Moreover, abdominal fat percentages responded quadratically (p<0.024) to the three doses, and abdominal skinfolds responded linearly (p = 0.003) and quadratically (p<0.021), but not for liver weight percentages.

### Xylo-oligosaccharides and lipid metabolism

The lipid metabolism is shown in Table 3. At 76, 80 and 84 wk, serum concentrations of cholesterol and triglycerides were also decreased (p<0.001) in the XOS treatments by 17.9%, 16.7%, and 17.5%, respectively; triglycerides were decreased by 26.9%, 27.0%, and 29.2%, respectively. There was no dose effect on the cholesterol, but with the increasing doses of XOS, linear (p = 0.035) and quadratic (p = 0.042) decreases were found on triglycerides.

### Xylo-oligosaccharides and folliculogenesis

**Figure 2:**
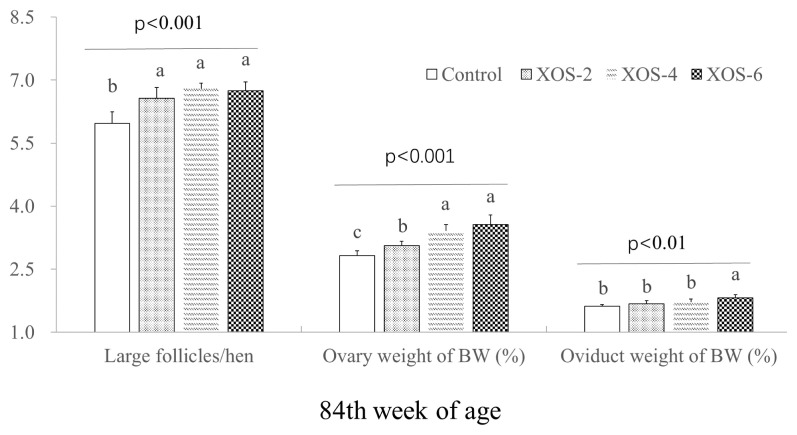
Effect of xylo-oligosaccharides (XOS) on large follicle number (LFN) and weight percentages of ovary and oviduct of Hy-Line hens at 84 wk of age. XOS-2, XOS-4, and XOS-6, containing XOS at 2.0, 4.0, and 6.0 mg/kg of diet. p<0.01 and p<0.001, treatment effect by Turkey’s-b test.

The dietary XOS influenced follicle numbers and weight percentages of ovary and oviduct (Figure 2). At 84 wk, hens with XOS supplementation at the three doses increased (p<0.05) the number of large follicles by 0.73 follicle/hen and the percentage of ovary weight by 18.6%. The oviduct percentage of XOS-6 was greater (p<0.05) than that of the control.

Additionally, a dose effect (p<0.05) was found on LH between XOS-2 and XOS-6 at 84 wk. FSH levels in XOS-4 and XOS-6 were higher (p<0.05) than XOS-2 at the last week. Ovary weight percentages of the middle and high doses of XOS were more pronounced (p<0.05) than XOS-2. The oviduct weight percentage of XOS-6 was higher (p<0.05) than that of XOS treatments.

**Figure 3:**
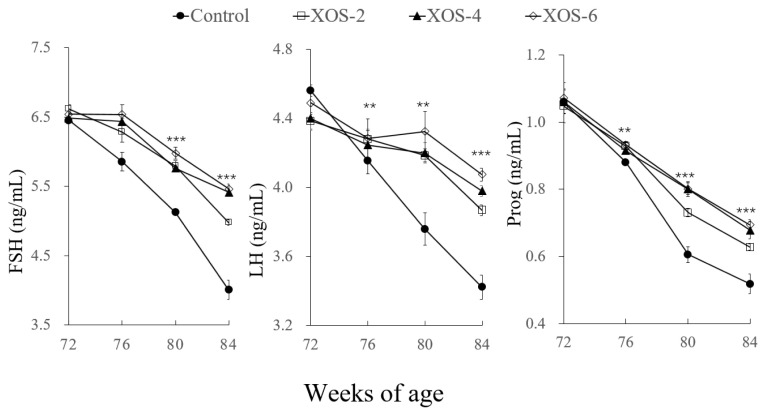
Effect of xylo-oligosaccharides (XOS) on serum reproductive hormones of Hy-Line hens. FSH, follicle-stimulating hormone; LH, luteinizing hormone; Prog, progesterone. XOS-2, XOS-4, and XOS-6, containing XOS at 2.0, 4.0, and 6.0 mg/kg of diet. ** p<0.01 and *** p<0.001, treatment effect by Turkey’s-b test.

For serum reproductive hormones (Figure 3), there were significant treatment effects on LH at 80 (p<0.01) and 84 wk (p<0.001); FSH and Prog at 76 (p<0.01), 80 (p<0.001), and 84 wk (p<0.001). Compared to the control, levels of LH at the three XOS treatments were higher by 12.7% and 16.1% at 80 and 84 wk, respectively; FSH and Prog were greater by 14.0% to 28.7%, respectively.

### Xylo-oligosaccharides and adipokines

**Figure 4:**
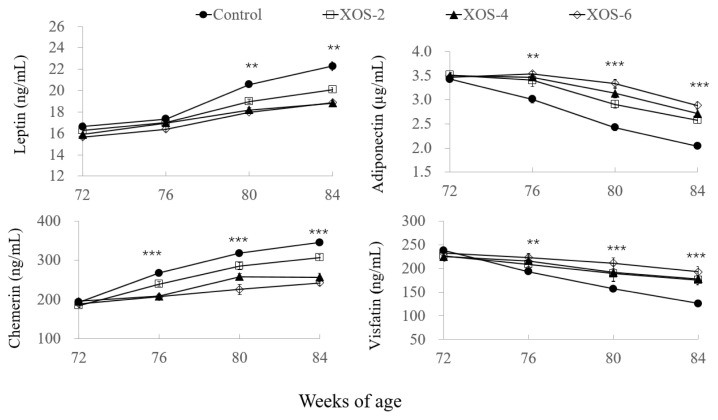
Effect of xylo-oligosaccharides (XOS) on serum adipokines of Hy-Line hens. XOS-2, XOS-4, and XOS-6, containing XOS at 2.0, 4.0, and 6.0 mg/kg of diet. ** p<0.01 and *** p<0.001, treatment effect by Turkey’s-b test.

In Figure 4, in the XOS diets at 80 and 84 wk, leptin levels were decreased (p = 0.001) by 10.5% and 13.6%, respectively, compared to the control. At 76, 80, and 84 wk of age, the dietary treatment influenced (p<0.01) the serum levels of adiponectin, chemerin, and visfatin. Compared to the control at the three age points, levels of adiponectin were increased (p<0.05) by 15.3%, 29.05%, and 33.8% and visfatin by 11.8%, 25.8%, and 44.0%, but chemerin levels were decreased (p< 0.05) by 18.4%, 19.4%, and 22.1%, respectively.

## DISCUSSION

In the present study, there were no statistical differences in feed intake and mortality with the dietary treatments throughout the trial (data not shown), and the daily feed provided at a constant amount of 108 g/hen avoided the interference of manipulated changes of feed amounts with egg production. The hens after the peak of reproduction were prone to accumulating body fat if they had access to feed *ad libitum*. Moreover, according to the local practice, if the egg laying rate starts dropping for three straight days, the feed ration will be correspondingly decreased; otherwise, the birds will have more and more body fat and weight, which will inevitably lead to a rapid egg laying drop and an early cull.

The hens at the late cycle of reproduction supplemented with XOS increased egg production, indicating that the dietary XOS can attenuate reproductive recession of older hens. The significant differences of egg-laying rates between the control and XOS treatments occurred firstly at 81 wk, and rapidly widened the gap from this time point, implying that aging hens were more susceptible to the XOS. Indeed, this did not occur when young laying hens were fed with XOS at 28 to 36 wk of age [15,16]. Whether this is mainly due to young hens having a less proneness to body fat deposition deserves further study. Importantly, the increased egg-laying rates led to a greater accumulated egg mass which finally reached a difference of 131 g/hen at 84 wk, which would result in an appreciable extra income for intensive farms.

Hens supplemented with dietary XOS had more follicles than the control, indicating that the XOS can improve the fecundity of hens, which was also consistent with the increased egg-laying rates. High circulating levels of reproductive hormones are a prerequisite for ensuring sustainably laying eggs. XOS is a functional feed fiber with potential to improve the reproductive efficiency of farm animals [17]. Indeed, the XOS supplementation in the present study beneficially affected the reproductive hormones LH, FSH, and Prog. Dietary factors such as apple pectic oligosaccharides are capable of influencing the follicle number and reproductive hormones in broiler breeders [18] and XOS modulated sterol excretion of hamsters [2]. However, literature is unavailable about the effect of XOS on follicular development and the secretion of reproductive hormones, and thus, more studies are needed.

The XOS decreased the body fat deposition in the present study. In practice, the reproductive duration of hens, even for the same genetic strain, always exhibits a tremendous variation from about 70 to 100 wk of age among layer enterprises, especially in developing countries. This coupled with the feeding cost from pullet period, causes a considerable profit difference, but this is also an opportunity for the improvement in feeding management [19,20]. An increased body fat deposition and thereby lipotoxicity to some extent is considered as the culprit leading to a rapid degradation in the laying capacity [7]. XOS blocking excessive adiposity in the liver, abdomen, and subcutis is a substantially effective approach in improving fecundity and is an alternative to feed restriction, a generally recommended nutritional countermeasure for reproductive recession [9,21]. In the present study, hens with greater fecundity had less body fat. But there is no literature reporting the action mode of dietary XOS on adiposity and fecundity. Additionally, the relationship between XOS and lipotoxicity of old hens is an interesting topic for the future study.

The addition of XOS in the present study also improved lipid metabolism by decreasing serum cholesterol and triglycerides. This is consistent with the study that XOS enhanced the excretion of neutral and acidic sterols, and promoted the production of SCFAs via changing the gut microbiota composition of hamsters [2]. During the growing and fattening period, XOS decreased the level of 1,7-heptane diamine and increased the acetic acid, straight-chain fatty acids, and total SCFAs concentrations in the intestinal contents of pigs [3]. In hens, information about the XOS effect on lipid profile is very limited, and the present study suggests that XOS improves lipid metabolism, which further affects the fecundity of hens.

The investigation into adipokines further explained the effect of XOS on lipid metabolism and body fat deposition. Adipokines, such as leptin, adiponectin, chemerin, and visfatin, are secreted by adipocytes and serve as modulators of nutritional state with reproductive activities [22]. Leptin is involved in the secretion of gonadotropins and ovarian steroidogenesis, and normal leptin levels promote ovarian granulosa and theca cell functions and oocyte maturation. However, the study of the obesity epidemic has elucidated leptin resistance pathways, with too much leptin leading to infertility [23]. In the present study, the XOS diets showed more stable serum levels of leptin from 72 to 84 wk, whereas its level in the control was increased at 80 and 84 wk. Similar results were found in other oligosaccharides. Dietary apple pectic oligosaccharide decreased serum leptin of hens with low laying rates but had no effect on hens with average laying rates [18]. The combination of green tea extract with isomalto-oligosaccharides modulated high fat diet induced adiposity, lipid accumulation, and leptin levels [24]. Fructooligosaccharides improved insulin and leptin sensitivities [25]. It is noted that under conditions of balanced nutrition, the secretion of leptin is timed and regulated within a narrow level range that optimizes its trophic effects, and the XOS in the present study may also contribute to the trophic effect of leptin, which needs further exploration.

Adiponectin can promote insulin sensitivity and regulate glucose and fatty acid catabolism, while chemerin is considered as an inhibitor of insulin signaling and glucose catabolism. Besides these established functions, adiponectin is known to promote gonadal activities, while chemerin exerts antigonadal actions [26]. The contrary effects of adiponectin and chemerin on the reproductive function were also observed in the present study that hens with high laying rates in the XOS treatments showed higher adiponectin and lower chemerin than hens with low laying rates in the control.

Visfatin regulates follicular growth, maturation of oocytes, dominance and selection of follicle and ovulation in the ovary [27], and this is proven by the results of present study that hens with supplemental XOS had more follicles and higher visfatin than the control. Unfortunately, literature is very limited about the effect of oligosaccharides on these adipokines. Only two studies showed that chitosan oligosaccharide supplementation reduced glucose absorption but increased plasma adiponectin levels in humans and rats [28]. Anyway, the present study first reported the effect of oligosaccharides on the serum adipokines pertaining to reproduction in female animals. Still, much regarding the XOS and adipokines has to be explored and brought forward in order to prolong durations of reproductive years in farm animals.

## CONCLUSION

Dietary XOS increased egg production by regulating ovary function, body fat, serum reproductive hormones, lipids, and adipokines of hens. The dose levels of XOS at 2, 4, and 6 g/kg did not have a significant effect on egg production. The results suggest that XOS as a widely and cheaply available additive can be added in the diet of aged animals to improve fecundity by counteracting adipotoxicity and enhancing productive signal factors.

## Figures and Tables

**Table 1:** Composition and nutrient level of basal diet (air-dry basis)

Items	Content (%)
Ingredient	
Corn	68.0
Soybean meal	14.4
Corn gluten	3.5
DL-Met (98%)	0.2
L-Lys (98%)	0.2
Salt	0.4
Soybean oil	1.5
Di-calcium phosphate	1.0
Limestone	9.8
Premix^1)^	1.0
Nutrient	
ME (Mcal/kg)^2)^	2.80
Crude protein	14.55
Ca	3.67
Non-phytate P	0.35
Met	0.41
Lys	0.75
Met+Cys	0.65
Crude fat	2.84
Crude fiber	1.97

1) Provides (per kilogram of diet): vitamin A (retinyl acetate), 8,000 IU; cholecalciferol, 1,600 IU; vitamin E (DL-α-tocopheryl acetate), 5 IU; vitamin K, 0.5 mg; riboflavin, 2.5 mg; D-pantothenic acid, 2.2 mg; niacin, 20 mg; pyridoxine, 3.0 mg; biotin, 0.10 mg; folic acid, 0.25 mg; vitamin B_12_, 0.004 mg; choline, 500 mg; manganese (MnSO_4_), 60 mg; iodine (NaI), 0.35 mg; iron (FeSO_4_), 60 mg; copper (CuSO_4_), 8 mg; zinc (ZnSO_4_), 80 mg; and selenium (Na_2_ SeO_3_), 0.30 mg.

2) ME, metabolizable energy, was calculated by Chinese Feed Database (2014, 25th ed) and others were measured.

**Table 2:** Effect of dietary XOS on egg mass and adipose deposition of Hy-Line hens

Item	Control	XOS (g/kg of diet)			SEM	p-value		
	
		2.0	4.0	6.0		T	L	Q
Egg mass								
73 to 76 wk (g/hen)	1,552	1,572	1,576	1,574	9.78	0.295	0.877	0.808
73 to 80 wk (g/hen)	3,038	3,069	3,069	3,083	15.21	0.243	0.515	0.717
73 to 84 wk (g/hen)	4,364^b^	4,478^a^	4,498^a^	4,509^a^	20.02	<0.001	0.269	0.852
Adipose deposition at 84 wk								
Liver weight (% of BW)	2.85^a^	2.43^ab^	2.39^ab^	2.32^b^	0.129	0.041	0.616	0.928
Abdominal fat (% of BW)	3.06^a^	2.52^bc^	2.65^b^	2.30^c^	0.071	<0.001	0.063	0.024
Abdominal skinfold (mm)	8.64^a^	7.10^b^	5.96^c^	6.09^c^	0.226	<0.001	0.003	0.021

XOS, xylo-oligosaccharides; SEM, standard error of the mean; T, treatment effect by Turkey’s-b test; L and Q, linear and quadratic, dose trend effect of XOS at 2.0, 4.0 and 6.0 mg/kg by polynomial contrasts; BW, body weight.

a–c Means in a row with different superscripts are significantly different (p<0.05).

**Table 3:** Effect of dietary XOS on serum lipid profiles of Hy-Line hens

Item	Control	XOS (g/kg of diet)			SEM	p-value		
	
		2.0	4.0	6.0		T	L	Q
Cholesterol (mmol/L)								
76 wk	8.95^a^	7.37^b^	7.32^b^	7.35^b^	0.221	<0.001	0.741	0.702
80 wk	9.38^a^	7.84^b^	7.81^b^	7.79^b^	0.302	<0.001	0.236	0.869
84 wk	9.81^a^	8.20^b^	8.16^b^	7.92^b^	0.297	<0.001	0.335	0.851
Triglyceride (mmol/L)								
76 wk	4.53^a^	3.24^b^	3.51^b^	3.19^b^	0.115	<0.001	0.644	0.183
80 wk	4.64^a^	3.50^b^	3.44^b^	3.22^b^	0.176	<0.001	0.035	0.339
84 wk	4.86^a^	3.53^b^	3.51^b^	3.28^c^	0.101	<0.001	0.042	0.412

XOS, xylo-oligosaccharides; SEM, standard error of the mean; T, treatment effect by Turkey’s-b test; L and Q, linear and quadratic, dose trend effect of XOS at 2.0, 4.0, and 6.0 mg/kg by polynomial contrasts.

a–c Means in a row with different superscripts are significantly different (p<0.05).

## References

[1] Lensu S, Pariyani R, Mäkinen E (2020). Prebiotic xylo-oligosaccharides ameliorate high-fat-diet-induced hepatic steatosis in rats. Nutrients.

[2] Abdo AAA, Zhang C, Lin Y (2021). Xylo-oligosaccharides ameliorate high cholesterol diet induced hypercholesterolemia and modulate sterol excretion and gut microbiota in hamsters. J Funct Foods.

[3] Pan J, Yin J, Zhang K (2019). Dietary xylo-oligosaccharide supplementation alters gut microbial composition and activity in pigs according to age and dose. AMB Express.

[4] Nicolucci AC, Reimer RA (2017). Prebiotics as a modulator of gut microbiota in paediatric obesity. Pediatr Obes.

[5] Lim SM, Kim E, Shin JH (2018). Xylobiose prevents high-fat diet induced mice obesity by suppressing mesenteric fat deposition and metabolic dysregulation. Molecules.

[6] Walzem RL, Chen SE (2014). Obesity-induced dysfunctions in female reproduction: lessons from birds and mammals. Adv Nutr.

[7] Amiri M, Tehrani FR (2020). Potential adverse effects of female and male obesity on fertility: a narrative review. Int J Endocrinol Metab.

[8] Chen SE, McMurtry JP, Walzem RL (2006). Overfeeding-induced ovarian dysfunction in broiler breeder hens is associated with lipotoxicity. Poult Sci.

[9] Dai H, Lv Z, Hu C (2020). Alpha-lipoic acid improves the reproduction performance of breeder hens during the late egg-laying period. J Anim Physiol Anim Nutr.

[10] Dupont J, Pollet-Villard X, Reverchon M, Mellouk N, Levy R (2015). Adipokines in human reproduction. Horm Mol Biol Clin Investig.

[11] Tan Y, Luo Y, Wang J, Liu N (2022). Effect of dietary tetramethylpyrazine on egg production, nutrient retention and cecal bacterial diversity in aged laying hens. Braz J Poult Sci.

[12] Liu N, Lin L, Wang JQ, Zhang FK, Wang JP (2019). Tetramethylpyrazine supplementation reduced Salmonella Typhimurium load and inflammatory response in broilers. Poult Sci.

[13] Li DD, Ding X, Zhang K (2017). Effects of dietary xylooligosaccharides on the performance, egg quality, nutrient digestibility and plasma parameters of laying hens. Anim Feed Sci Technol.

[14] Liu N, Ding K, Wang J, Deng Q, Gu K, Wang J (2018). Effects of lactic acid bacteria and smectite after aflatoxin B1 challenge on the growth performance, nutrient digestibility and blood parameters of broilers. J Anim Physiol Anim Nutr.

[15] Li XY, Xiao L, He G (2017). Xylo-oligosaccharides. China National Standard.

[16] Ding XM, Li D, Bai S (2018). Effect of dietary xylooligosaccharides on intestinal characteristics, gut microbiota, cecal short-chain fatty acids, and plasma immune parameters of laying hens. Poult Sci.

[17] Tian M, Chen J, Liu J (2020). Dietary fiber and microbiota interaction regulates sow metabolism and reproductive performance. Anim Nutr.

[18] Wang J, Zhang C, Zhao S (2021). Dietary apple pectic oligosaccharide improves reproductive performance, antioxidant capacity, and ovary function of broiler breeders. Poult Sci.

[19] Altahat E, Al-Sharafat A, Altarawneh M (2012). Factors affecting profitability of layer hens enterprises. Am J Agric Biol Sci.

[20] Hastang, Sirajuddin SN, Ikrar MS (2019). Level of competitiveness of laying hens. IOP Conference Series: Earth Environ Sci.

[21] Zhao S, Zhang K, Ding X (2019). The impact of dietary supplementation of different feed additives on performances of broiler breeders characterized by different egg-laying rate. Poult Sci.

[22] Mellouk N, Ramé C, Barbe A (2018). Chicken is a useful model to investigate the role of adipokines in metabolic and reproductive diseases. Int J Endocrinol.

[23] Childs GV, Odle AK, MacNicol MC, MacNicol AM (2021). The importance of leptin to reproduction. Endocrinology.

[24] Singh DP, Singh J, Boparai RK (2017). Isomalto-oligosaccharides, a prebiotic, functionally augment green tea effects against high fat diet-induced metabolic alterations via preventing gut dysbacteriosis in mice. Pharmacol Res.

[25] Shinoki A, Hara H (2011). Dietary fructo-oligosaccharides improve insulin sensitivity along with the suppression of adipocytokine secretion from mesenteric fat cells in rats. Br J Nutr.

[26] Singh A, Choubey M, Bora P, Krishna A (2018). Adiponectin and chemerin: Contrary adipokines in regulating reproduction and metabolic disorders. Reprod Sci.

[27] Park BK, Park MJ, Kim HG (2020). Role of visfatin in restoration of ovarian aging and fertility in the mouse aged 18 months. Reprod Sci.

[28] Lee JY, Kim TY, Kang H (2021). Anti-obesity and anti-adipogenic effects of chitosan oligosaccharide (GO2KA1) in SD rats and in 3T3-L1 preadipocytes models. Molecules.

